# Supplementary material for: Variation in Antibiotic Treatment Failure Outcome Definitions in Randomised Trials and Observational Studies of Antibiotic Prescribing Strategies: A Systematic Review and Narrative Synthesis
Source: Antibiotics (Basel). 2022 May 6;11(5):627. doi: 10.3390/antibiotics11050627 (PMC9137992; doi:10.3390/antibiotics11050627)
Supplement: Supplementary file 1 [file antibiotics-11-00627-s001.zip › antibiotics-1662121-supplementary.pdf]

Variation in antibiotic treatment failure outcome definitions in randomised trials and observational studies of antibiotic prescribing strategies: A systematic review and narrative synthesis

Supplementary material

Table S1. Description of antibiotic treatment failure components used

**Component category**	**Component details**	**Number of studies**
Prescription change	Change to different antibiotic	10
Prescription change	Another prescription for the same antibiotic	2
Prescription change	New prescription for the same or a different antibiotic within 10 days	1
Prescription change	Change to intravenous antibiotic	3
Prescription change	Addition of a second antibiotic	3
Prescription change	Increase in antibiotic duration to >14 days	2
Prescription change	Physician initiated change of antibiotic 3-30 days after index antibiotic	1
Prescription change	Index antibiotic changed due to new infection/serious adverse event	1
Prescription change	Second antibiotic within 14 days of index antibiotic	1
Prescription change	Reconsultation & same-day antibiotic within14 days of index event	2
Prescription change	New prescription 14-30 days from index event	1
Prescription change	Additional/change in antibiotic within 28 days	1
Prescription change	Treatment switch & new diagnosis within 7 days	1
Prescription change	Additional outpatient visit requiring antibiotic within 42 days	1
Prescription change	New antibiotic prescription with 4-28 days	1
Prescription change	New antibiotic and diagnostic code 14-28 days from index event	2
Prescription change	Change in antibiotics <72 hours after index event	1
Clinical condition	Any signs of WHO defined severe pneumonia	1
Clinical condition	Recurrence of fever within 5-14 days	1
Clinical condition	Withdrawal from trial at 48 hours or days 5-14	1
Clinical condition	Development of serious adverse events at 48 hours or days 5-14	1
Clinical condition	Tachypnoea at 48 hours or days 5-14	1
Clinical condition	Persistence of fever at 48 hours or days 5-14	1
Clinical condition	Fast breathing	2
Clinical condition	No clinical improvement/worsening on day 3	1
Clinical condition	Increase in respiratory rate between days 2-5	1
Clinical condition	Lower chest indrawing	3
Clinical condition	Clinical relapse during days 3-30	1
Clinical condition	WHO ICMI danger signs	4
Clinical condition	Death	7
Clinical condition	Persistence of symptoms	2
Clinical condition	Appearance of new symptoms	1
Clinical condition	Fever days 3-4	1

Clinical condition	Increase in erythema (>25%), swelling or tenderness days 3-4	1
Clinical condition	No decrease in erythema, swelling or tenderness days 8-10	1
Clinical condition	More than minimal erythema, swelling or tenderness	1
Clinical condition	New infection within 14 days of index event	1
Escalation of care	Emergency department visit	10
Escalation of care	Hospital admission	16
Escalation of care	Specialist referral	3
Escalation of care	Surgical intervention	1
